# Our New York Letter

**Published:** 1888-04

**Authors:** 


### Correspondence.

### OUR NEW YORK LETTER.

THE MEDICAL ASPECTS OF JOSEF HOFMANN'S CASE—DUPUYTREN'S FINGER CONTRACTION—THE NEW LOOMIS LABORATORY—A PHYSICIAN IN TROUBLE.

*To the Editors Atlanta Medical and Surgical Journal:*

Considerable interest has lately been aroused among physicians concerning the medical aspect of the case of Josef Hofmann, the nine year old musical prodigy. During the winter this boy has been in the habit of giving public piano concerts before enormous audiences, playing the most difficult, serious compositions, and also improvising on given themes. He continued to perform in this way almost nightly for a time until the Society for the Prevention of Cruelty to Children interfered and put a stop to the concerts. The mayor then appointed an examining committee of medical men to investigate the young musician's case, and these gentlemen finally reported that the amount of his work should be lessened. This was done and his performances were limited to four a week, but the strain has evidently proved too severe for his strength and he is again resting at the advice of his physician, Dr. Simon Baruch. On behalf of the boy's manager, a board of examiners have now been appointed, consisting of Doctors Lewis A. Sayre, Austin Flint and Allen McLane Hamilton. These physicians report that the boy is of a somewhat nervous temperament, but has no organic trouble. With the following exceptions they regard him well and strong: His temperature has at times been a little above 100° F. and his pulse has occasionally been over 100 and slightly irregular. They consider that at times his work has been too laborious, but think it would be safe to reduce the number of his concerts to one a week, and that he should not perform more than four times weekly. Dr. Baruch agrees with these gentlemen that Josef Hofmann has no organic trouble, but from his elevated temperature, irregular pulse and other symptoms, he considers that his patient has severe nervous derangement brought on by mental and physical strain. This boy has also suffered from incontinence of urine during and after his performances, and taking all the symptoms into consideration, Dr. Baruch has insisted that his patient must have complete rest, and he has therefore refused to sign the report of the above-mentioned physicians. As the non-appearance in public of this musical wonder means a heavy loss to the management, a considerable stir has been made over the affair, but the general opinion among physicians seems to be that Dr. Baruch is right, and that what the boy most needs is complete repose.

A very interesting communication on "Dupuytren's Finger Contraction" was recently read by Dr. Robert Abbe before the surgical section of the Academy of Medicine. He considers this infrequent disease to be essentially a hyperplasia of the palmar aponeurosis, involving the areolar connective tissue and sometimes the skin also. Dr. Abbe does not believe that gout causes this trouble, as is generally supposed, and his opinions are based upon an observation of forty cases. He explains this disease somewhat as follows : It originates from a slight traumatism of the hand, which by its peripheral irritation causes a spinal impression. A reflex influence to the hand then occurs, causing hyperæmia and a growth of new contractile tissue. Joint troubles somewhat like rheumatism may occur and the contractions of the bands may cause neuralgias, and also a reflection of these symptoms to the same parts of the opposite hand. One hand is first affected and the other usually not until several years later. Scar tissue in any part of the body, he said, might cause neuralgia in distant parts, which entirely disappeared on excising the scar. Associated with Dupuytren's contraction he has observed various neuroses, which the patient often considers to be another disease, but which in reality originate from the palmar lesion and disappear upon its removal. A patient under his care had suffered from backache for years, for which all forms of local and general treatment were tried and found useless. An operation upon his contracted fingers permanently cured him.

The histories of numerous cases were then read. Most of the patients had suffered for a number of years, and many attributed the trouble to slight causes, as a tight finger-ring, carrying a whip in the palm of the hand, or to similar things. Division of the contracting band gave instant and permanent relief to all the symptoms. The pain, when present, he considers due to the sensory nerve branches being involved in the new contractile tissue. His method of operating is as follows: Esmarch's bandage being applied to the limb, cocaine anæsthesia is employed and the band either excised, or multiple transverse incisions are made. A gauze dressing prepared with a 1–1,000 sublimate solution is then used, but splints are not necessary as a rule. In about ten days the parts are generally healed, and stretching, if called for, may then be resorted to.

A few days ago the new Loomis Laboratory was formally opened, and for about three hours the building was crowded with interested visitors, who were received by the college dean, Dr. Charles Inslee Pardee. This institution is probably one of the most complete of the kind. The first floor is fitted up for the practical study of *materia medica* and physics. On the second floor is the chemical department, including a laboratory for toxicology, which contains all the latest forms of apparatus that may be required in medico-legal cases. The other floors are devoted to bacteriology, pathology, biology and physiology. The name of the donor of this magnificent laboratory is still withheld.

A physician of this city was recently arrested and tried for taking a patient with small-pox to the hospital by means of the elevated railroad. In explaining his position, the doctor said he was unable to find a policeman and considered that the best interests of the public would be served by getting his patient into the hospital as soon as possible. The judge appeared to be fairly satisfied with this statement and discharged the prisoner after imposing a fine of one dollar. A. F.

*New York, March 8, 1888.*

